# Supplementary material for: Stimulation and Repair of Peripheral Nerves Using Bioadhesive Graft‐Antenna
Source: Adv Sci (Weinh). 2019 Apr 3;6(11):1801212. doi: 10.1002/advs.201801212 (PMC6548953; doi:10.1002/advs.201801212)
Supplement: Supplementary file 1 — Supplementary [file ADVS-6-1801212-s002.pdf]

Copyright WILEY-VCH Verlag GmbH & Co. KGaA, 69469 Weinheim, Germany, 2019.

ADVANCED SCIENCE
Open Access

Supporting Information

for *Adv. Sci.,* DOI: 10.1002/advs.201801212

Stimulation and Repair of Peripheral Nerves Using Bioadhesive Graft-Antenna

*Ashour Sliow, Zhi Ma, Gaetano Gargiulo, David Mahns, Damia Mawad, Paul Breen, Marcus Stoodley, Jessica Houang, Rhiannon Kuchel, Giuseppe C. Tettamanzi, Richard D. Tilley, Samuel J. Frost, John Morley, Leonardo Longo, and Antonio Lauto**

Supporting Information

Stimulation and Repair of Peripheral Nerves Using Bioadhesive Graft-Antenna

*Ashour Sliow, Zhi Ma, Gaetano Gargiulo, David Mahns, Damia Mawad, Paul Breen, Marcus Stoodley, Jessica Houang, Rhiannon Kuchel, Giuseppe Tettamanzi, Richard D Tilley, Samuel J Frost, John Morley, Leonardo Longo and Antonio Lauto****

Supplementary 1.1 (Surgery)

Rodents were anaesthetized using 2% Isofluorane in 100% oxygen using a standard anaesthetic machine. Under sterile conditions, a 3-4 cm skin incision was made between the ischial tuberosity and the knee joint of the right leg. The plane between the gluteus maximus and biceps femoris muscles was identified and dissected using blunt dissection to expose ~1.5 cm of the sciatic nerve proximal its distal trifurcation. Under an Olympus operating microscope (1–40x), the nerve (diameter ~1 mm) was freed by dissecting surrounding connective tissue using a microscissor; care was taken to minimize nerve handling. The electrical response of the muscle was measured using a purpose-built AC coupled differential amplifier (100x gain, 1 hz High pass filter) and recorded using a digital to analog converter (Model 1401, Cambridge Electronic Design, UK). The recording electrode was placed in the rectus femoris muscle, the reference electrode was positioned to the adjacent tissues while the ground electrode was fixed to the leg skin of the rat. The relative position of electrodes was carefully measured with callipers for further data analysis.

Supplementary 1.2 (Ammeter)

A bespoke ammeter designed around the low noise instrumentation amplifier (INA118, Texas Instruments INC., USA) was used to measure the current flow induced in the loop antenna. The small current flow is transformed into a small voltage using a sampling resistor (nominal conversion factor: 1 μA = 1.2 mV). This resistor (1.2 Ω) is connected directly between the

INA118 inputs and generates a voltage directly proportional to the current flow that is amplified by a factor of 1000 [V/V]. The amplified signal is directly acquired by the Powerlab system that it is used to record the action potential. The reference terminal of the INA118 is directly connected to the general grounding, which includes the rat and the full instrumentation, to avoid creation of ground loops. The bespoke ammeter is calibrated using a precise current source obtained with a 1/3 of REF200 (Texas Instruments INC., USA). Calibration can be verified by switching the current input of the sampling resistor to the calibrated constant current (100 µA) contained inside the REF200.

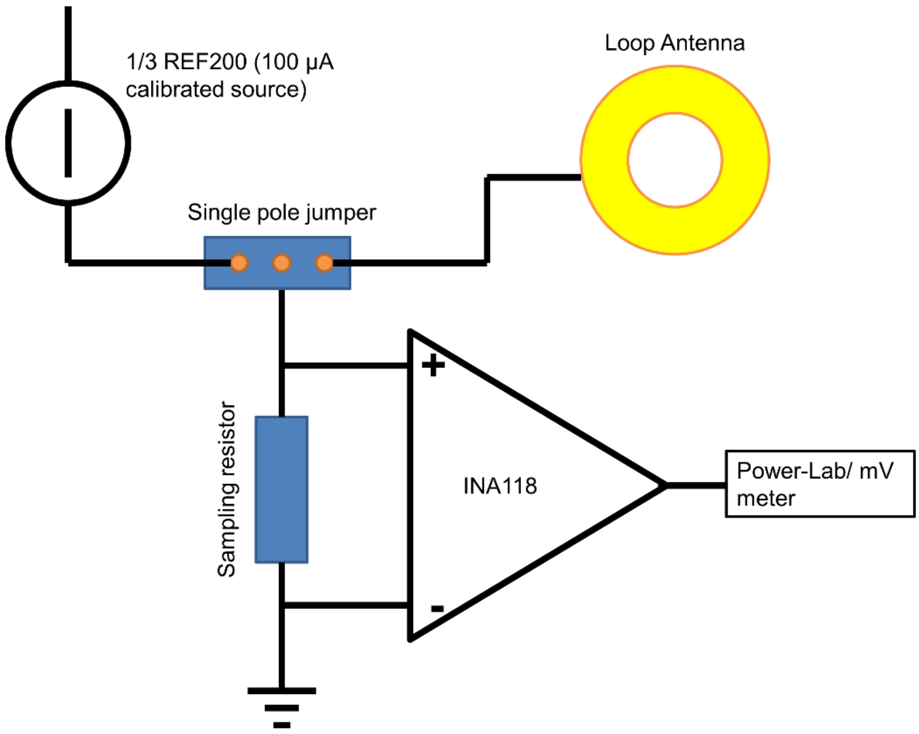

Simplified diagram of the bespoke ammeter (see text).

Supplementary 1.3 (Graft-Antenna)

The adhesive film was prepared using the method published earlier by our group [28, 29]. Briefly, medium molecular weight chitosan (598 cps viscosity, 81% deacetylation; Sigma-Aldrich, Sydney, NSW, Australia) was dissolved at a concentration of 1.7% (w/v) in deionized water that contained 2% (v/v) acetic acid and 0.01% (w/v) rose bengal. The viscous

solution was stirred for 14 days at room temperature (~25 °C) in the dark to avoid photo-bleaching of rose bengal. Insoluble matter was removed by centrifuging the rose bengal-chitosan solution at 3270×g for an hour. The collected supernatant was spread uniformly (~1.2 ml over ~12 cm^2) on a dry and sterile Perspex plate at room temperature. The solution was allowed to dry over 3 weeks which caused ~90% water content loss, forming a thin film which did not dissolve in water [30]. The rose bengal-chitosan film was carefully detached from the plate avoiding damage and small rectangular sections (~5x5 mm) were cut with scissors. An Emitech K550X gold coater (Quorum Emitech, East Sussex, England) sputtered a strip of gold onto the adhesive using a filter paper template. The chitosan adhesive was placed underneath the template and a gold strip was deposited with a width of 0.8 ± 0.1 mm and thickness of 50-80 nm. When this adhesive is placed around the nerve, the gold strip becomes a loop antenna that can receive electromagnetic radiation. The adhesive graft-antennas were stored in a sterile plastic box and kept in the dark at room temperature to avoid dye photobleaching.

Supplementary 1.4 (Histology)

Before euthanasia, sciatic nerves were exposed at the site of operation and inspected for neuroma formation, tissue adhesion and uncharacteristic inflammation. The nerves were then harvested in ~15 mm lengths and fixed in 5% paraformaldehyde solution in 0.1 M phosphate buffer for 24 hours at ~4 °C. Nerves were serially dehydrated in ethanol and embedded in paraffin. Transverse sections of 5–10 mm thickness were made 5 mm proximal and distal to the adhesive site. Samples were stained with Luxol Fast Blue to visualize myelinated axons and Haematoxylin and Eosin (H&E) to evaluate any adverse effect on nerves due to the TMS stimulation. Histological slides were scanned and analysed using an Aperio XT Slide Scanner (Aperio, Vista, CA, USA). Images were numbered and their identity concealed during

analysis, which was carried out on ~55% of the cross-sectional area of the operated and non-operated (contralateral) nerves.

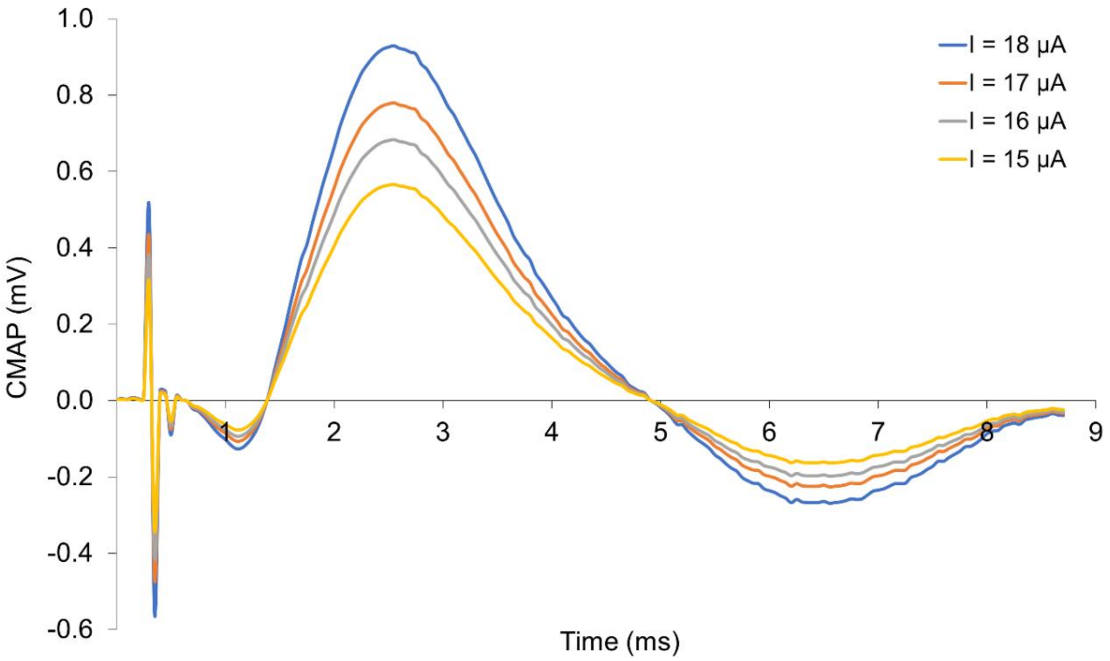

Figure S1. DC stimulation of sciatic nerves. Compound muscle action potentials (CMAPs) triggered in healthy sciatic nerves (controls) by a DC stimulator at different current levels. The signal amplitude decreases sharply from ~0.9 to ~0.5 mV when the current drops from 18 μA to 15 μA, respectively (n = 3).

Table S1. CMAP and copper loop antenna voltage. Legend. *CMAP + Oscilloscope,* Compound Muscle Action Potential amplitude measured when the loop antenna around the nerve is powered by the TMS. The loop antenna is connected to the oscilloscope. *CMAP*, CMAP amplitude measured when the loop antenna around the nerve is powered by the TMS without oscilloscope connection. *CNAP + oscilloscope*, amplitude of Compound Nerve Action Potential with antenna connected to oscilloscope. *CNAP*, amplitude of Compound Nerve Action Potential without oscilloscope connection. *Loop Voltage*, voltage induced in the copper loop antenna by the TMS. Three independent experiments were performed in each group (n = 3); 120 measures were averaged for the amplitude and voltage values in each experiment.

	**Amplitude (mV)**	**Loop Voltage (mV)**
CMAP + Oscilloscope	0.67 ± 0.09	10.49 ± 0.07
CMAP	0.70 ± 0.07	-
CNAP + Oscilloscope	0.33 ± 0.06	10.49 ± 0.06
CNAP	0.33 ± 0.05	-

Table S2. Histomorphometric results (uncut nerves). Histomorphometric results of the nerves 12 weeks after graft-antenna implantation. The TMS stimulated the nerves via the graft-antenna once a week for 1 hour (1 pulse/sec) at 60% B_{max} (~0.72 T). The analysis was performed on ~55% of the total cross-sectional area of nerves (n = 5).

	**Proximal**	**Distal**	**Control**
**Myelinated Axon Count**	1858 ± 75	1846 ± 81	1849 ± 71
**Nerve Fiber Diameter (µm)**	5.9 ± 1.6	5.8 ± 1.7	5.6 ± 1.5
**Axon Diameter (µm)**	3.6 ± 1.5	3.6 ± 1.6	3.5 ± 1.5
**Myelin Thickness (µm)**	2.1 ± 0.8	2.1 ± 0.7	2.1 ± 0.6
**Nerve Area (%)**	54 ± 7	55 ± 9	55 ± 7
